# Low‐pitch peripheral systolic murmur associated with pulmonary embolism in the acute phase: a report of two cases

**DOI:** 10.1002/ccr3.1411

**Published:** 2018-02-14

**Authors:** Ahmadou Musa Jingi, Sylvie Ndongo Amougou, Bonaventure Jemea, Christian Ngongang Ouankou, Armelle Foutko, Narcisse Assene Ateba, Clovis Nkoke

**Affiliations:** ^1^ Department of Internal Medicine and Specialties Faculty of Medicine and Biomedical Sciences University of Yaounde 1 Yaounde Cameroon; ^2^ Intensive Care Unit University Teaching Hospital of Yaounde Yaounde Cameroon; ^3^ Department of Surgery Faculty of Medicine and Biomedical Sciences University of Yaounde Yaounde Cameroon

**Keywords:** Low‐pitch, periphery, pulmonary embolism, systolic murmur

## Abstract

Acute pulmonary embolism with significant right ventricular strain could be associated with a low‐pitch peripheral systolic murmur radiating to the axillae.

## Introduction

Pulmonary embolism (PE) is frequent and is often underdiagnosed and thus, not treated accordingly as most cases are diagnosed at autopsy 1, 2, 3, 4. When suspected, contrast‐enhanced computed tomography (CT) is not readily available or affordable to confirm the diagnosis in low‐income settings, where if available, is located only in tertiary hospitals in the main cities 4, 5, 6. Probability scores of PE have been developed from clinical data, but these need to be improved so as to capture many cases in the absence of sophisticated imaging studies. This is particularly useful for the clinician in low‐income settings where diagnostic tests are not readily available and affordable. We report two cases of peripheral low‐pitch systolic ejection murmur at the base of the heart in two patients in whom there was no evidence of valvular stenosis on echocardiography in the context of confirmed pulmonary embolism on CT scan. Such murmurs have been reported elsewhere, and its occurrence is not widely recognized 7, 8, 9. We highlight the importance of detecting this murmur, which we think is clinically important in low‐income settings. The patients were hospitalized and managed in the Intensive Care Unit (ICU) of the University Teaching Hospital of Yaounde–Cameroon, sub‐Saharan Africa (SSA) in March 2016. We report this work in accordance with the standards for reporting Case Report (CARE) guidelines.

## Case Presentation

### Case number 1

It is a 35‐year‐old woman who was admitted in the ICU for acute onset NYHA grade 3 dyspnea evolving since 4 days. The dyspnea began 4 days after an eight‐hour flight in the economic class.

Her past medical history was remarkable for acute Cor Pulmonale diagnosed 3 months before her present admission. She was regularly treated with acenocoumarol 4 mg daily. She admitted being compliant to treatment but was not regularly checked for efficacy of anticoagulation. She stopped using intramuscular contraceptive about 10 months ago. She reported two spontaneous abortions before the twelfth week of gestation when she was aged eighteen. She has been experiencing menstrual bleeding since 9 days. She denied tobacco use, and consumed alcohol occasionally. She denied any chronic disease.

**Figure 1:**
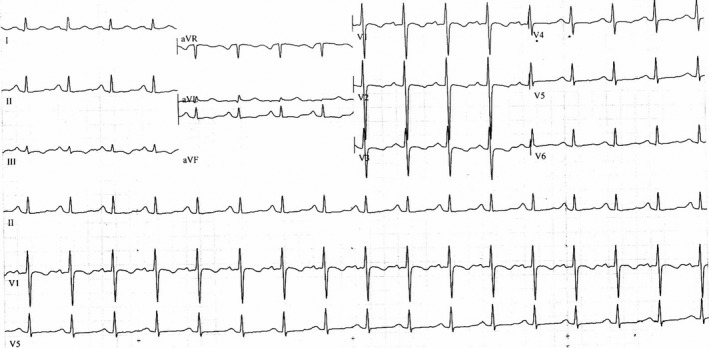
ECG of case number 1 shows regular sinus rhythm with inverted T‐waves in leads V1–V4, aVF, D2, and D3. Flattened T‐waves in V5, V6.

**Figure 2:**
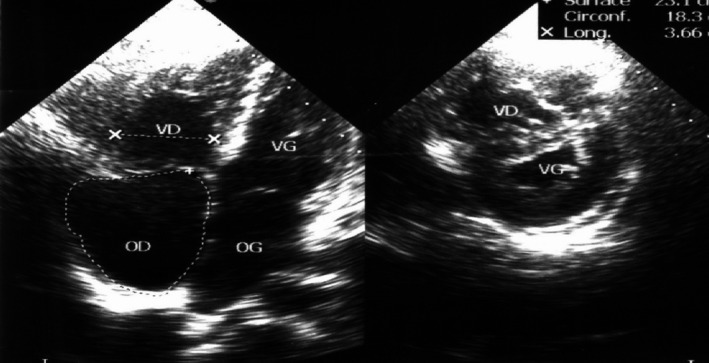
Echocardiogram of case number 1 shows markedly dilated right cavities with mass effect on the left cavities. The pulmonary systolic pressure was estimated at 85 mmHg.

On examination, she was not acutely ill‐looking and in no acute distress. Her blood pressure was 100/60 mmHg with a heart rate of 120 beats per minute. Her respiratory rate was 32 cycles per minute, and her oxygen saturation was 91% on room air. She was markedly obese. The heart sounds were audible, with accentuation of the second sound in the pulmonary area, and a gallop rhythm over the tricuspid area. There was a low‐pitch systolic ejection murmur (grade 2/6) in the aortic area, with a maximum intensity (grade 3/6) heard in the intersection of the second intercostal space and the right midclavicle line during a few seconds of apnea. The murmur was radiating to the right axilla but there was no radiation to the neck vessels or to the back. No systolic murmur was heard in the pulmonary area. The lungs were clear to auscultation. There were no clinical signs of deep venous thrombosis nor signs of right ventricular failure. The rest of the clinical examination was unremarkable. The diagnosis of probable recurrence of pulmonary embolism was made based on intermediate pretest clinical probability (modified Geneva score of 8). A contrast‐enhanced CT pulmonary scan showed multiple proximal and segmental filling defects of the right pulmonary arteries. ECG showed regular sinus tachycardia, with inverted T‐waves in lead V1–V4, D2, aVF, and D3 (Fig. 1). Echocardiography showed markedly dilated right heart chambers with mass effect on the left chambers, a pulmonary artery systolic pressure of 85 mmHg, and normal aortic valves (Fig. 2). The rest of the echocardiography was unremarkable. Pending investigation for the etiology, she was treated with 12‐hourly subcutaneous low molecular weight heparin (LMWH) at a dose of 1 mg/kg, and acenocoumarol 4 mg daily. Her INR at this dose was 1.85. Her clinical evolution was uneventful, and she was discharged after 7 days of hospitalization with persistence of the low‐pitch systolic ejection murmur. This murmur was not audible 4 weeks after her discharge.

### Case number 2

**Figure 3:**
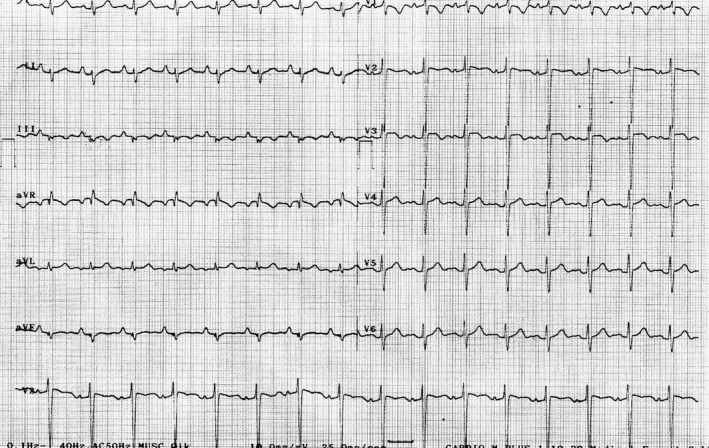
ECG of case number 2 shows regular sinus Tachycardia, right atrial enlargement, inverted T‐waves in leads V1–V3, and S1Q3T3 pattern.

**Figure 4:**
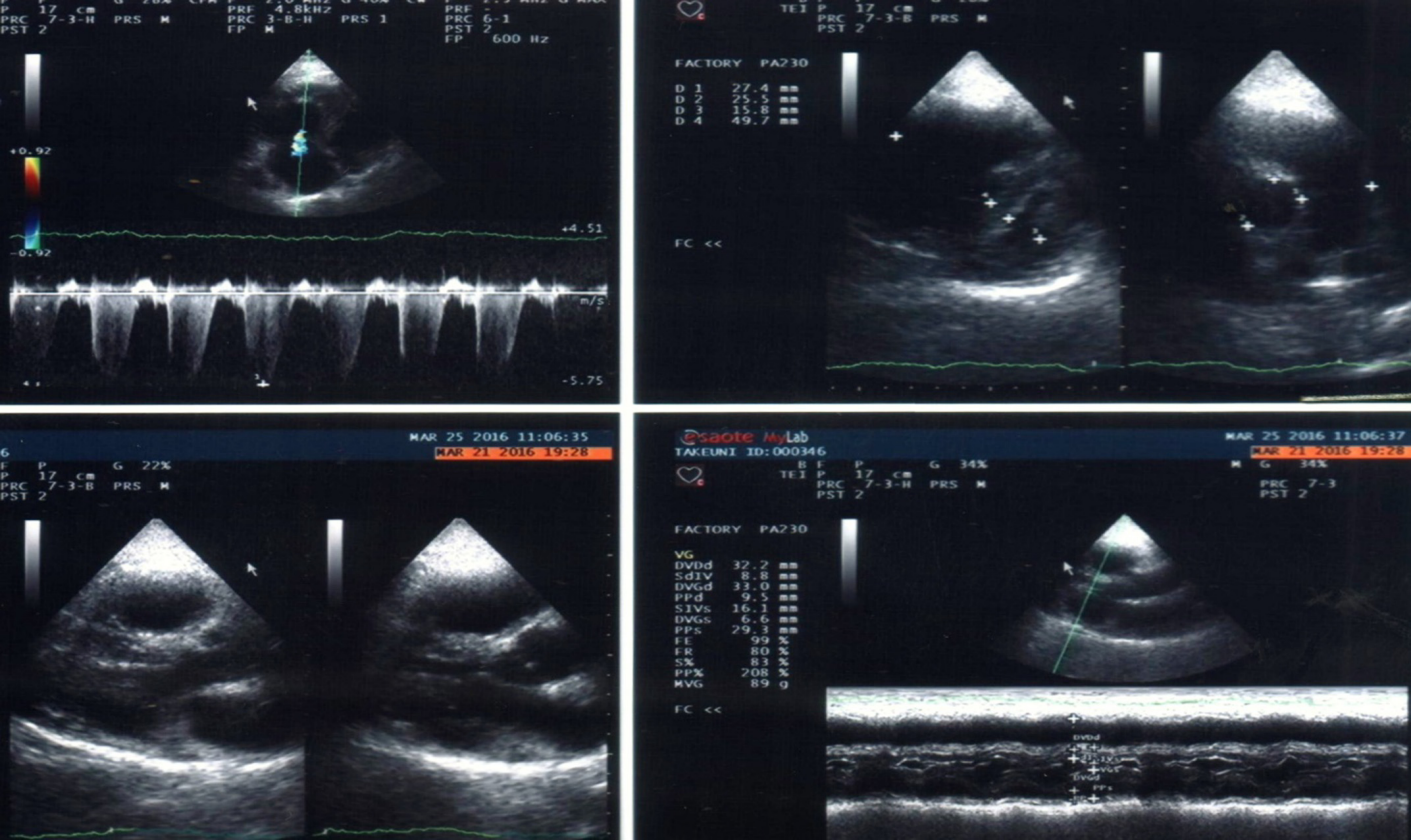
Echocardiogram of case number 2 shows markedly dilated right cavities with marked mass effect on the left cavities. The pulmonary systolic pressure was estimated at 148 mmHg.

It is a 58‐year‐old man, who was admitted in the ICU for acute onset NYHA grade 3 exertional dyspnea, cough productive of mucoid sputum, and nonspecific chest pain, evolving since 7 days. His past medical history was remarkable for arterial hypertension. He stopped smoking cigarettes 27 years ago. On admission, his blood pressure was 180/110 mmHg with a heart rate of 110 beats per minute. His respiratory rate was 36 per minute, with oxygen saturation of 96% with 3 L/min of oxygen. He was obese and afebrile. His heart sounds were audible, with accentuation of the second component in the pulmonary area, and a S3 gallop rhythm over the tricuspid area. There was a low‐pitch systolic ejection murmur (grade 2/6) heard in the intersection of the left second intercostal space and the left mid‐clavicle line during a few seconds of apnea. This murmur was not heard beyond this intersection. There was no radiation of the murmur to the back. No systolic murmur was heard in the aortic area. A low‐pitch murmur (grade 3/6) was heard in the right axillary region, at the intersection of the midaxillary line and the fourth and fifth intercostal space. There were no crepitations on lung auscultation. There were no clinical signs of deep venous thrombosis nor signs of right ventricular failure. The rest of the clinical examination was unremarkable. The diagnosis of pulmonary embolism was made based on an intermediate pretest probability (Wells score 4.5). A contrast‐enhanced CT pulmonary scan showed multiple filling defects of the pulmonary arteries bilaterally, with areas of lung consolidation. ECG showed regular sinus tachycardia, right atrial enlargement, inverted T‐waves in leads V1–V3, and an S1Q3T3 pattern (Fig. 3). Echocardiography showed dilated right heart chambers with a raised pulmonary pressure of 148 mmHg and normal pulmonary valves (Fig. 4). Pending investigation for the etiology, he was treated with 12‐hourly subcutaneous LMWH at a dose of 1 mg/kg and acenocoumarol 4 mg daily with an uneventful recovery. The low‐pitch murmurs persisted at the time of discharge, which became inaudible 2 weeks after discharge.

## Discussion

Low‐pitch systolic murmur heard at the base of the heart with normal heart valves on echocardiography in the context of pulmonary embolism has been reported but not known by many medical practitioners 7, 8, 9, 10. We report a similar low‐pitch systolic murmur heard at the base of the heart in two patients in sub‐Saharan Africa, with contrast‐enhanced CT scan confirmed pulmonary embolism and normal heart valves at echocardiography. This might have clinical implications in the diagnosis of pulmonary embolism in the acute phase in low‐income settings where contrast‐enhanced CT scan is not readily available or affordable, or when a patient with suspected pulmonary embolism is not stable enough to undergo the test. The diagnostic accuracy of this murmur needs to be further investigated.

In the first case, the systolic murmur contrasted with that of aortic stenosis, which is known to be a high pitch systolic murmur that radiates to the neck vessels and associated left ventricular structural and functional anomaly. The systolic murmur in this patient was low‐pitched, and was best heard in the intersection of the right second intercostal space and the right midclavicle line, and radiated toward the right axilla. In the absence of a structural aortic valvular stenosis or intercavity communication on echocardiography, this low‐pitch systolic murmur can be attributed to turbulent flow across the partially obstructive thrombus in the right pulmonary artery. In the second case, the low‐pitch systolic murmur at the base region of the heart contrasted with that of pulmonary valvular stenosis in that, it was not best heard at the pulmonary area but at the intersection of the left second intercostal space and the midclavicle line. Also, it did not radiate to the back region. The murmur was also heard in the right axillary region, an unusual location for any heart murmur. In the absence of a structural pulmonary or aortic valve stenosis or intercavity communication on echocardiography, this murmur can also be attributed to turbulent flow across the partially obstructive thrombus in the left and right pulmonary arteries. The differences noticed between the cases could be explained by differences in the size of the thrombi. A large thrombus or thrombus with significant obstruction will lead to significant turbulence and a louder murmur than a small thrombus with partial obstruction.

This report has some weaknesses. We could not record the murmur or study its waveform because we lacked the necessary equipment, such as a phonocardiograph. This murmur can easily be confused with breath sounds, thus requiring auscultation during a brief period of breath holding. We describe this murmur in stable patients in no acute distress, who can cooperate and thus hold their breath. Breath holding is not possible in a patient in acute distress and raises ethical issues. In the absence of respiratory distress, the clinician in search of this murmur should also observe apnea at the same time as the patient. Also, in preparing this report, we had technical difficulties in acquiring good quality images of the CT scans. Despite these limitations, the semiology of this murmur associated with PE is the key element of this report.

## Conclusion

Acute Pulmonary Embolism with significant right ventricular strain could be associated with a low‐pitch peripheral systolic (LOPES) murmur radiating to the axillae. The diagnostic performance of LOPES in the context of acute pulmonary embolism needs to be studied.

## Ethical Considerations

The patients gave consent for their case to be published, so as to help ameliorate the diagnosis and treatment of PE in low‐income settings.

## Authorship

AMJ: participated in patient care and drafted the manuscript. CN: drafted the manuscript and participated in patient care. CNO: participated in patient care and read the final draft. AF: participated in patient care and read the final draft. NAA: participated in patient care and read the final draft. SAN: participated in patient care and read the final draft. BJ: participated in patient care and read the final draft. All authors approved of the final version and the decision to publish the work.

## Conflict of Interest

We declare no conflict of Interest.

## References

[1] Shimi, M. , M. Allouche , H. Ben Ahmed , B. Zoghlemi , F. Gloulou , M. Ben Khelil , et al. 2014 Sudden death due to pulmonary embolism in north Tunisia: 37 cases study. Tunis Méd. 92:610–614. 25860675

[2] Touze, J. E. , G. Moncany , A. Amonkou , G. Cailleau , A. Monnier , M. Kacou , et al. 1985 Pulmonary thromboembolic diseases in Ivory Coast (apropos of 13 cases). Méd. Trop. (Mars) 45:43–46. 2985907

[3] Datta, B. N. , K. Ramesh , and B. Bhusnurmath . 1986 Autopsy incidence of pulmonary vascular episodes. A study of 218 cases. Angiology 37:744–750. 3767065 10.1177/000331978603701008

[4] Ogeng'o, J. A. , M. M. Obimbo , B. O. Olabu , P. M. Gatonga , and D. Ong'era . 2011 Pulmonary thromboembolism in an East African tertiary referral hospital. J. Thromb. Thrombolysis 32:386–391. 21674133 10.1007/s11239-011-0607-4

[5] Hantous‐Zannad, S. , S. Esseghaier , I. Ridène , A. Zidi , K. Ben Romdhane , I. Baccouche , et al. 2010 Acute pulmonary embolism: epidemiologic and tomodensitometric study. Tunis Méd. 88:880–884. 21136353

[6] Tambe, J. , B. Moifo , E. Fongang , E. Guegang , and A. G. Juimo . 2012 Acute pulmonary embolism in the era of multi‐detector CT: a reality in sub‐Saharan Africa. BMC Med. Imaging 12:31. 23072500 10.1186/1471-2342-12-31 PMC3485620

[7] Godycki‐Ćwirko, M. , and A. Bratkowska . 2011 An 89‐year‐old patient with acquired murmur associated with pulmonary embolism. Arch. Med. Sci. 7:902–904. 22291839 10.5114/aoms.2011.25569 PMC3258820

[8] Okada, R. D. , and G. A. Ewy . 1983 The acquired nonprecordial peripheral pulmonary murmur of pulmonary embolism. Chest 83:762–766. 6839817 10.1378/chest.83.5.762

[9] ZuWallack, R. L. , J. P. Liss , and B. Lahiri . 1976 Acquired continuous murmur associated with acute pulmonary thromboembolism. Chest 70:557–559. 975962 10.1378/chest.70.4.557

[10] Cohen, S. I. , H. Hecht , J. Cantor , and E. Morkin . 1975 Flow murmur associated with partial occlusion of the right pulmonary artery. Am. Heart J. 90:376–379. 1163429 10.1016/0002-8703(75)90328-2

